# “Go with the Flo”: Conducting rapid research on prenatal stress following Hurricane Florence as participant observers

**DOI:** 10.3389/fsoc.2022.957127

**Published:** 2022-11-24

**Authors:** Michaela Howells, Kelsey Dancause

**Affiliations:** ^1^Department of Anthropology, University of North Carolina Wilmington, Wilmington, NC, United States; ^2^Exercise Science, Université du Québec à Montréal, Montreal, QC, Canada

**Keywords:** rapid research, natural disaster, extreme weather, participant observation, pregnancy, Hurricane Florence, stress

## Abstract

In this article, we explore the challenges of conceptualizing, designing, and establishing a rapid research agenda as a local researcher following a disaster. We share what we learned while developing and implementing this rapid study and explore the challenges shaped by time pressures, our local context, and resource availability. We identify four core challenges, experienced conducting rapid research, and provide suggestions to overcome these challenges. Our goal is to provide insight to undergraduates, graduate students, and professionals who are considering rapid research inside or outside their own communities.

## Introduction

Natural disasters are increasing in frequency and intensity [Intergovernmental Panel on Climate Change (IPCC), 2022]. Triggered by climate change, these experiences have global ramifications to physical, social, and community well-being [World Health Organization (WHO), 2014; United States Environmental Protection Agency (EPA), 2017; United States Department of Health and Human Services (HHS), 2021]. Social science research helps to capture the nuanced experiences of those affected, which can inform future prevention and intervention strategies and provide critical feedback to city planners and local and state governments (Peek et al., 2020). However, research in disaster areas runs the risk of taking resources in short supply that would be used for citizens of the affected area (e.g., housing), and requires creation of partnerships at a time of intense strain on systems (Gaillard and Peek, 2019). As a result, researchers living in affected areas are in a particularly strong position to conduct rapid research associated with the event. In this article, we explore the benefits and challenges local researchers encounter when conducting research following disasters. We provide an honest and frank case study of conducting research in our own community and discuss the unforeseen challenges and barriers to this research approach. We hope this can provide insight to students and professionals assessing their preparedness to take on disaster response research in their communities.

Community based knowledge provides key advantages for research. This knowledge can come from multiple sources including effective community partnerships, participant observation, and from relationships with community members who are also researchers (Gaillard and Peek, 2019). Investigators that are from communities being studied may bring both the perspective of a researcher and a community member to their projects. Because they live, work, and play within the community on a regular basis, they bring additional connections and perspectives to the research through their lived experiences. This is not to argue that communities are homogenous, or that significant privilege doesn't separate a researcher and the members of the community they study. However, it does provide an additional insight into the context of the area and supports rich community connections.

Less is written on the strain that these studies can have on the researcher themselves, and the unintended impact that can have on conducting their research (Mukherji et al., 2014). Although we are trained researchers who have worked in challenging conditions around the globe, the impact of experiencing a natural disaster and trying to establish a research program presented some unforeseen complications for our team. We are trained to think that we should be able to function as researchers regardless of conditions. However, the personal trauma of a disaster affecting your city, being evacuated and displaced for an extended period of time, balancing an emerging multifaceted research project with teaching and other professional responsibilities, as well as these effects on collaborators and networks, requires additional attention and planning.

Here, we share our experience trying to balance these conflicting challenges. Our core academic research team consists of a local (Howells) and non-local (Dancause) researcher. In this paper, we use auto-ethnography to explore Howells' experience of being a local researcher simultaneously experiencing the impacts of disaster and developing rapid research. Our reflexive process occurred in the months after the event and was principally through discussions with collaborators and colleagues, both informally and at conferences where we presented preliminary study results. Some of the difficulties associated with our rapid research mirrored those experience by our colleagues during the COVID-19 pandemic, which helped us to more concretely identify and describe key challenges. Furthermore, Howells used social media to document challenges, observations, and updates in the weeks after the disaster. These brief but frequent notes of observations provided a means to organize and document thoughts and experiences as a disaster survivor and a researcher, and provided a perspective of others' experiences and reactions through their comments.

Both Howells and Dancause have experience in analyzing prenatal stress, including in potentially vulnerable samples such as in low and middle-income countries and among socially disadvantaged communities that experience persistent perinatal health burdens such as prematurity and low birthweight. Having seen the effects of prenatal stress in vulnerable samples, our individual and collaborative research programs are driven by a desire to draw attention to the importance of reducing health disparities and improving the environment for socially disadvantaged communities in general, including during and following disasters. Applying our expertise to develop a study in the Wilmington community was driven by a desire to make not only a scientific contribution, but also to amplify the voices of those affected by the disaster and to potentially draw attention to particular needs and targets to improve the environment for community members. This background in prenatal stress, health disparities, and with vulnerable samples affects our positionality and our approach to the research.

## Before the storm

Hurricanes are part of living on the US coastal southeast. Remnants from previous storms, warnings to ensure you have materials ready to evacuate, and reminders to stockpile batteries, canned goods, and drinking water in the case of a storm create a perpetual awareness of the possibility of disaster. However, this also results in a normalization of risk and coping mechanisms that defer those concerns to a later date. In the last week of August 2018, we received the first serious warnings of Hurricane Florence developing in the Atlantic. It was the second week of school at the University of North Carolina Wilmington where I (Howells) was in my third year of teaching as an Assistant Professor. In the coming days, it became evident that my husband and I would need to make a quick decision between sheltering in place through the storm or leaving before the roads were impassable. The forecasted severity of the storm combined with the privilege of having reliable transportation and multiple housing options resulted in us evacuating 400+ miles away to my family's home in Atlanta.

In Atlanta, we were safe, but watched with trepidation as the storm increased in intensity and moved with threatening speed toward our community. As the storm drew closer, the Wilmington community's social media platforms were filled with stories of people choosing to stay or leave, about those who had no choice, about jam packed roads out of town, and distressed people telling their stories. The Weather Channel centered its disaster narrative on Wilmington, and we were transfixed by the impending disaster.

As a researcher specializing in the impact of maternal stress and disparate access to health care, I was particularly drawn to those stories of pregnant people navigating the impending disaster and the uncertainty they were facing. As the storm creeped closer, I contacted my colleague Dancause in Montreal, who had completed significant pregnancy disaster work abroad both independently and as a member of the Stress in Pregnancy International Research Alliance (SPIRAL). Together, we initiated a collaborative study on maternal health and stress following the hurricane. We designed an empirical study to capture prenatal stress due to the storm and its impact on maternal and infant health—even as I was preparing for the impending storm.

The decision to initiate this study arose from wanting to use our research skills to contribute to the scientific literature on prenatal stress and perinatal health, but also to help tell the story of the Hurricane and help the Wilmington community. Although the study was an overall success, we faced multiple challenges in designing and implementing this rapid research. These included (1) misinterpreting the complexity of initiating a study when faced with personal trauma from the event, (2) misjudging the complexity involved in combining protocols with local collaborators who were also experiencing the stress of the event, (3) underestimating realistic delays in campus research support offices, (4) misinterpreting the strength of our community connections. In other words, we were well-intentioned but naïve.

## The storm hits

On September 14, 2018, Hurricane Florence made landfall and for 2 days produced record-breaking rainfall in North Carolina—up to 30 inches in an area already threatened by previous rains and sandy, ill-draining soil [National Weather Service (NWS), 2019]. This led to significant flooding, erosion, and destruction of property and infrastructure. As the storm passed over, scenes of destruction emerged. I watched people on TV paddle kayaks through the usually bustling downtown. A friend's destroyed home was being used by multiple media outlets as an example of the destruction. Several of the postcard perfect 100+ year old live oaks that lined the city streets were upended by the storm. Several buildings at the university—including residence halls and a science building—were irrevocably damaged. Power throughout parts of the city stayed off for weeks, and evacuated residents (including my family) were asked to stay away.

## The study

There is nothing like the helplessness you feel when you are watching your city struggle with a natural disaster and all you can do is wait for permission to return. During this time, we wrote and submitted a Quick Response Grant to the Natural Hazards Center, Boulder Colorado to support our work. We started developing a protocol combining interviews and questionnaires (in person and online) and framework for our material. Our study proposed to capture maternal stress prior to and following the storm. We developed a custom-made survey of experiences during and following the hurricane based on previous SPIRAL studies (King et al., 2015), and combined them with other measures of mediators and moderators of stress including sociodemographic characteristics, social support, and coping styles (Howells et al., 2020). The majority of these interviews were conducted in my campus office, however I did meet several women in mutually agreed upon areas around the city. I would describe the study and receive their informed consent. The majority chose to complete the questions themselves, and afterwards we would discuss their experiences. These ranged from the stress of their work hours being cut due to damage to their place of employment, to being bitten by snakes during the evacuation process. Many mentioned the distress of being evacuated and separated from their health care team.

In addition to these interviews, we collected maternal hair to measure cortisol in the months before and following the disaster. Hair cortisol provides a non-invasive measure of stress in the preceding months (D'Anna-Hernandez et al., 2011; Stalder et al., 2012). With a 4-cm section, we would be able to assess cortisol levels reflecting the 2 months following the disaster (the proximal 2 cm of hair) and the 1–2 month period before the disaster (the distal 1–2 cm of hair). The questionnaire and hair collection protocols that we developed were largely based on Dancause's past experience in disaster research in other settings. This represented an area where she could contribute to help advance the study, while I took on responsibility for identifying local resources and recruitment sites, communicating with collaborators, completing tasks related to my university review process such as Internal Review Board (IRB) applications, and tailoring the protocol to the local context. Her work and perspective throughout the process was also invaluable at ensuring that were not being mired in inconsequential details that did not support our end goals.

The rapid nature of the study combined with my own experiences of evacuation resulted in additional challenges in the development of the research. Both of us are trained in community participatory research and strive to tailor study objectives and data collection through discussions and feedback with community members, including potential study participants and stakeholders such as public health collaborators. This process of participatory research was less feasible following the disaster. The evacuation meant that meeting face-to-face with key stakeholders was not possible, and rapidly changing and uncertain conditions complicated engaging in discussions with community collaborators and potential participants. Furthermore, our data collection methods had to be adapted both in response to the need to act rapidly, and also to the burdens of participants experiencing difficult and uncertain conditions, who might not have the time or capacity to participate in a complex protocol. As such, we had to make difficult decisions about which information to prioritize and which to exclude from our data collection, with less feedback from community members than in our studies under “typical” conditions.

## After the storm—Returning home

Returning home was emotionally challenging. The roads were littered with destroyed trees and collapsed structures. Our home was thankfully intact, although the lack of power for over 2 weeks in humid southeastern North Carolina left a thin layer of mold over everything inside. I shopped for groceries away knowing our local grocery store shelves were functionally empty. I returned to campus and began the challenging process of addressing the psychological and educational needs of our students. There were multiple meetings, and workshops to support the reconfiguration of our classes and make up for the lost month of courses. Faculty had to pick up the pieces of our classes and make significant modifications to our syllabi—with full recognition of the trauma our students had experienced and continued to experience.

Students had lost a great deal of their stability. In many cases, their homes, belongings, and books were destroyed. Everyone knew someone who had lost everything. Although being back in the classroom felt like a step toward normalcy, it was also exhausting and required additional physical, psychological, and emotional labor. Those who recently transitioned their classes due to the COVID-19 pandemic have a strong understanding of this challenge (Adedoyin and Soykan, 2020). In addition, I was personally struggling being away from my spouse who works two hours away. The evacuation gave us precious and unusual time together and being apart intensified the emotional strain of the disaster. Although these challenges were not directly associated with rapid disaster-based research, they speak to the undercurrent of distractions and challenges facing researchers working in the field following a disaster.

## Conducting the work—A different kind of storm

### Collaborations, research permission, and funding

We were thankful to receive grant funding contingent on IRB (Internal Review Board, comparable with European Independent Ethics Committee) approval. We had started preparing our IRB application while the storm was still active. However, because the university was closed these offices were offline, and we focused our attentions elsewhere in the study preparations until they reopened.

By the time the system was online, we had added another collaborator who had ideas about embedding our study in a larger related study. This would expand the reach of our results and provide a strong comparative data set. We met multiple times to create study protocols and IRB applications that encapsulated both programs. We prioritized the IRB from this larger study because of the possibility of additional funds, personnel, and interdisciplinary reach. Part of the logic was that once the larger IRB was in place, we would be able to add or modify details specific to our study, maximizing our time investment and gaining approval for both protocols. I felt secure in this collaboration because the collaborator had completed significant disaster work in the global south and was connected within the local academic community. It felt powerful being part of a larger study, and I embraced the opportunity.

Unfortunately, merging the studies did not work as hoped. The larger study was unable to move forward as planned, and as such, trainings and data collection events that had been planned were canceled and we had no access to the student research assistants who were supposed to assist with data collection. Members of the research team associated with the larger study were facing their own challenges associated with the disaster, which affected the progress of both studies. Because we had all conducted similar research abroad, it hadn't occurred to me that our ability to perform as researchers could be impacted by our concurrent role as disaster survivors.

We had no provisions in the study to prepare for this situation, and it set our original team back both mentally and temporally. Furthermore, the IRB encapsulating both programs did not, upon close inspection, encompass the key aspects of our proposed work. We had to create and submit a new IRB to our university research office that was by then backlogged with requests and dealing with their own challenges of reopening following the storm. To credit this office, they were supportive and professional throughout, and provided timely feedback on our application.

During this time, we were lucky to be awarded a Quick Response Grant from the Natural Hazards Center. The grant office at my university was in the early stages of reopening and inundated with requests. The delays regarding assessment and approval of the use of funds resulted in a loss of an additional two and a half weeks of data collection. I had not taken into account that my support offices would be overextended and that it would take additional time before the start of research. By the time we had permissions to launch our research from both offices, it was over months from the hurricane's landfall. Our project's novelty depended on being able to sample pregnant people's hair within a three month period to capture cortisol before and after the disaster. Unfortunately, these unexpected delays narrowed the number of people we could recruit. However, we were able to develop a picture of maternal stress with this hair (Howells et al., 2023).

### Community based work

A month into our data collection, we were invited to collaborate with a local health center focused on providing health services (including prenatal care) to underserved populations. This invitation stemmed from my pre-existing connections in the health community with nurse practitioners specializing in maternal health. This collaboration enabled us to connect with health care providers and pregnant people, and expanded our recruitment and reach. Health providers would do the initial check in with participants, and assess their interest in hearing more about the project. We would interview these participants before or after their normal check-ups. This helped diversify our participant pool and established a strong relationship moving forward. A minor challenge arose that the clinic times were scheduled during my teaching times. Both had been long established and there was no flexibility. I was able to attend the clinic for the first two hours and then would rely on my student research assistant to attend the remaining time.

Given the difficulty for students returning to class while recovering from the disaster, recruiting student research assistants with the capacity to take on additional responsibilities was more difficult than I had previously experienced. This limited our capacity to adapt to the hours of the clinic, to recruit participants, and to collect data as quickly as we had hoped.

My collaboration with the local health center was successful. However, in general I vastly overestimated my other community connections. Although I had an extensive local community network and was involved with multiple organizations, I realized the kind of relationships that are critical to this research were not ones I had in place. This was exacerbated by the effects of the disaster. Groups and organizations that would have made for strong partnerships were understandably focused on serving the needs of their clients and their employees.

If I had established stronger community research connections before the disaster, I would have been able to ensure the foundation was in place for our rapid response study. Even after having work extensively abroad, I misunderstood how challenging it would be to create relationships with health care teams and to navigate multiple levels of administration, different systems of integrating researchers into clinical activities, and different personnel interests and capabilities (in terms of time commitments and experience with research) across sites. We were already limited by the number of pregnant people in our study area and reaching them was hampered by not having relationships with the providers.

## Discussion

The contributions of rapid research and evaluation are undeniable (Oulahen et al., 2020). However, researchers initiating rapid studies following a disaster experience particular challenges associated with time and resource constraints. Local researchers have an unparallel opportunity to engage with their communities following a disaster (Gaillard and Peek, 2019). However, these researchers face many of the same challenges as external researchers in addition to novel ones. In this article, we developed a case study considering the experience of implementing a novel rapid research project as local researchers. This was meant to be a frank and personal examination of the lessons learned by our research team while designing and implementing our study.

Being a local researcher resulted in unanticipated complications in the implementations of our work. These were associated with time pressures, local contexts, and access to resources. The core components discussed in this article were (1) misinterpreting the complexity of initiating a study when faced with personal trauma from the event, (2) misjudging the complexity involved in combining protocols with local collaborators who were also experiencing the stress of the event, (3) underestimating realistic delays in campus research support offices, and finally, (4) misinterpreting the strength of our community connections. In Table 1 we explore these unanticipated challenges and provide suggestions for addressing these before and during the initiation of rapid research.

**Table 1:** Addressing unanticipated challenges associated with rapid research as a local researcher.

**Issue**	**Explanation**	**Recommendations**
Personal trauma from the event	Researchers are also impacted by disaster events but may not recognize the effects of trauma on their work. Disaster may disrupt typical work making their job harder.	• Collaborating with non-local researchers who can help maintain perspective. • Psychological first aid training prior to events [e.g., completing psychological first aid courses through American Red Cross, 2017]. • Taking breaks, talking with loved ones, journaling, exercising, paying attention to changes in appetite and motivation.
Misjudging the complexity involved in combining protocols with local collaborators who were also experiencing the stress of the event	In rapid research quick collaborations need to be established. There is less time to closely evaluate the fit of study protocols or the goals and capacities of team members who might themselves be experiencing stress and trauma due to the event.	• When possible, build on collaborations developed before the study's initiation. • When possible, build larger and more diverse teams of both local and non-local collaborators who can take on extra tasks or help find new solutions when other members are experiencing particularly stressful conditions. • Develop a memo of understanding that documents honest and upfront discussions of expectations including dialog between team members (Holgate, 2012).
Underestimating realistic delays in campus research support offices	If a disaster impacts the area, it may disrupt the support offices academic researchers depend on to conduct their work.	• Contact related offices (research permissions, grant offices) early in the process to ensure they are aware of what you are planning. • If offices are closed take advantage of any online portholes that may remain open. By submitting research permission (IRB) paperwork before they open it may help prioritize it on their return. • Plan on the process taking longer than usual. Offices may be short staffed due to personal traumas and dislocations. They also may be inundated with requests when they reopen. It is expected that their turn around times would be disrupted.
Misinterpreting the strength of community connections	Local researchers may over interpret their collaborations with community partners.	• Complete cultural competence and cultural humility training (frequently offered for free from governments or universities). • Develop authentic collaborations that forefront community engagement at all stages starting before the disaster (Swann et al., 2020). • Be engaged with community groups and organizations prior to the disaster. • Ask for help openly and honestly from existing community collaborators at all stages of research. • Recognize that this will likely be a slow process needed to build trust and move through all of the permissions and authorizations.

Many of the challenges outlined in Table 1 are relevant even in non-disaster situations. Furthermore, the challenge of tailoring a protocol to resource constraints and to favor and retain participation is likely familiar to many researchers regardless of the setting. However, recovering from the disaster coupled with the need to act rapidly amplified the effects of these challenges on our study design, data collection, and outreach compared to our research experiences in non-disaster settings. Although our study protocol was designed with the challenges of rapid research and the disaster setting in mind, we experienced complications in launching the study, recruitment, and data collection that were exacerbated by the strain associated with disaster recovery for me, my colleagues, university administration, and students.

Despite these challenges, as a local researcher conducting rapid disaster-based research following Hurricane Florence, I benefited from having established housing and transportation during a time of severe shortages. Our research also benefited from being associated with the local university and health care center and was tailored to the specific needs of our community. In addition, it meant our research team could incorporate these tasks into their typical work week without taking a leave of absence or suspending their academic positions. Finally, our project was strengthened by a strong collaboration between local and non-local researchers and community collaborators. This relationship provided a healthy balance during a challenging time and the possibility to distribute tasks, where possible, among team members.

Many of the suggestions we propose are relevant in other situations that affect our capacity to conduct our studies as usual, such as during the COVID-19 pandemic, which disrupted campus support services and data collection procedures in many institutions and created a personal emotional strain for members of research teams. Developing techniques to deal with stress and trauma, building larger and more diverse teams to better enable researchers to adapt quickly in the face of changing local conditions and restrictions, remaining in close contact with support offices, and actively engaging with community collaborators are relevant to launching research studies under difficult and uncertain conditions. We hope that by sharing our experiences, challenges, successes, and lessons learned we will be able support other professionals and students in successfully designing and implementing their rapid research.

## Data availability statement

The original contributions presented in the study are included in the article/supplementary material, further inquiries can be directed to the corresponding author/s.

## Ethics statement

The studies involving human participants were reviewed and approved by University of North Carolina Wilmington (19-0086). The patients/participants provided their written informed consent to participate in this study.

## Author contributions

MH and KD conceptualized, designed, wrote, and received grants that supported this research. MH and KD collaboratively wrote this manuscript. All authors contributed to the article and approved the submitted version.

## Funding

Funding was provided by the Natural Hazards Center Quick Response Grant and the University of North Carolina Wilmington SURCA.

## Conflict of interest

The authors declare that the research was conducted in the absence of any commercial or financial relationships that could be construed as a potential conflict of interest.

## Publisher's note

All claims expressed in this article are solely those of the authors and do not necessarily represent those of their affiliated organizations, or those of the publisher, the editors and the reviewers. Any product that may be evaluated in this article, or claim that may be made by its manufacturer, is not guaranteed or endorsed by the publisher.
